# Mental Health Burden Among People Living With HIV/AIDS in Southern Vietnam: Prevalence and Novel Risk Factors for Depression and Anxiety

**DOI:** 10.21315/mjms-11-2024-909

**Published:** 2025-06-30

**Authors:** Yen Thi Hoai Phan, Thao Thi Ngoc Nguyen, Vu Hoang Anh Nguyen, Yen Phi Nguyen, Phuong Thi Be Nguyen, Thanh Nguyen Ai Tran

**Affiliations:** 1 Faculty of Public Health, University of Medicine and Pharmacy at Ho Chi Minh City, Vietnam; 2 Department of Psychosomatic Medicine, Thu Duc City Hospital, Ho Chi Minh City, Vietnam; 3 Center of Quality Control for Medical Laboratory, University of Medicine and Pharmacy at Ho Chi Minh City, Vietnam; 4 Department of Internal Medicine, Thu Duc City Hospital, Ho Chi Minh City, Vietnam; 5 Board of Directors, Thu Duc City Hospital, Ho Chi Minh City, Vietnam

**Keywords:** anxiety disorder, depression, PHQ-9, GAD-7, PLWHA

## Abstract

**Background:**

Anxiety and depression are prevalent among people living with HIV/AIDS (PLWHA), with significantly higher rates than in the general population. As HIV has become a manageable chronic condition through antiretroviral therapy (ART), understanding its mental health impact is essential. This study examines the prevalence and key factors associated with anxiety and depression among PLWHA at an ART clinic in South Vietnam’s Thu Duc City Hospital.

**Methods:**

A descriptive cross-sectional study was conducted on 369 PLWHA being treated at the ART clinic at Thu Duc City Hospital from March to May 2024. Convenience sampling was used to select the participants, all age 18 and up, who were interviewed face-to-face using a structured questionnaire that included PHQ-9 and GAD-7.

**Results:**

Of the study’s 369 participants, 82.4% were male, with most between ages 19 and 39 (71.8%). Anxiety prevalence was 29.3% (95% CI: 24.6–34.1), while depression was reported by 23.0% of participants (95% CI: 18.8–27.6). Significant factors associated with anxiety disorders included experiencing side effects during ART treatment (AOR = 5.92, 95% CI: 1.54–22.7, *P* = 0.01) and high levels of HIV stigma (AOR = 2.51, 95% CI: 1.45–4.33, *P* = 0.001). Depression was associated significantly with severe anxiety (AOR = 101.61, 95% CI: 11.67–884.28, *P* < 0.001) and moderate anxiety (AOR = 58.06, 95% CI: 22.27–151.40, *P* < 0.001).

**Conclusion:**

This study highlights the critical need to address mental health challenges among PLWHA in South Vietnam, with an emphasis on the importance of reducing stigma, managing ART-related side effects and integrating mental health care into HIV treatment. The findings provide valuable insights for developing appropriate contextual interventions.

## Introduction

To this day, the World Health Organisation (WHO) views HIV/AIDS as a significant public health concern due to its ongoing transmission across countries worldwide. WHO reports revealed that in 2022, approximately 39 million people were living with HIV/AIDS (PLWHA) globally, 630,000 died from conditions related to HIV and about 1.3 million new infections were reported (1). Sub-Saharan Africa is the most severely affected region worldwide, with about 25.6 million cases, representing two-thirds of the global population living with HIV. The Asia-Pacific region follows, with approximately 3.9 million cases (2, 3). As of October 2022, Vietnam recorded 220,580 PLWHA, with 112,368 deaths attributed to the disease (4).

Despite extensive efforts, a complete cure for HIV remains out of reach, but the introduction of antiretroviral therapy (ART) has proven effective in suppressing HIV replication, significantly reducing HIV-related mortality and transforming HIV from a life-threatening disease to a manageable chronic condition (5). Since the implementation of ART treatment, the number of deaths due to HIV-related causes in 2022 decreased by approximately 51%, and the number of new cases dropped by 38% compared with 2010 (2, 3). To optimise treatment outcomes, mental health burdens among PLWHA need to be addressed, as these burdens are often significant and complex. Extant research indicates that PLWHA face a higher burden from mental health challenges due to a combination of factors: social and demographic conditions; psychological stigma; limited support networks; economic hardship; lower educational attainment; chronic health issues; substance use; difficulties with consistent medication adherence; side effects from ART treatments and changes in CD4 cell counts (6–10). These mental health issues can disrupt daily life significantly, impacting work, social relationships and family dynamics (11, 12).

Globally, depression and anxiety represent major mental health burdens for PLWHA, with prevalence rates ranging from 13% to 78% for depression and 19% to 38% for anxiety (8, 13–15). More than half of PLWHA experience anxiety and depression, which is higher than that of the general population (16, 17). Studies have indicated that PLWHA with depression and anxiety are associated with suicidal behaviour, low adherence to treatment, substance abuse and poor treatment outcomes (18–20).

As Vietnam progresses towards controlling HIV spread through the effective implementation of ART and widespread access to care, the mental health challenges faced by PLWHA require renewed attention. The transition from a life-threatening epidemic to a manageable chronic condition has reshaped the psychological and social realities of PLWHA (21). However, literature gaps remain in understanding the mental health burden in this new era, particularly in identifying emerging risk factors unique to Vietnam’s evolving socioeconomic and healthcare contexts. In Vietnam, while several studies have examined the prevalence of depression and anxiety among PLWHA, the specific factors impacting their mental health during treatment remain underexamined. Thu Duc City Hospital, a Grade I facility located at the intersection of major industrial provinces in South Vietnam, is part of the country’s newest city and a unique healthcare hub. With 2,500 PLWHA currently receiving care at its outpatient clinic, Thu Duc City Hospital provides a critical setting for examining mental health in this population, particularly as the region continues to see one of the highest rates of new HIV cases in the nation. This study aims to investigate not only the prevalence of anxiety and depression, but also novel, context-specific risk factors associated with these conditions among PLWHA. The findings will contribute to a more comprehensive understanding of mental health challenges among PLWHA and provide a foundation on which to develop targeted psychosocial and healthcare strategies to improve long-term treatment outcomes.

## Methods

### Study Area

This study was conducted from March to May 2024 at Thu Duc City Hospital, located in Thu Duc City, South Vietnam. As the newest city in Vietnam, Thu Duc City is positioned strategically as an industrial and technological hub, serving as a key intersection of several provinces in South Vietnam. Thu Duc City Hospital, a Grade I hospital, plays a central role in providing advanced medical services to this rapidly growing region. The hospital’s outpatient clinic currently cares for and treats approximately 2,500 PLWHA. Beyond offering ART, the clinic places a strong emphasis on psychological support and counselling, aiming to help patients navigate challenges, enhance their health and build resilience.

### Study Design and Population

A descriptive cross-sectional study design was chosen. The study’s sample comprised PLWHA receiving ART at Thu Duc City Hospital during the data collection period. Participants in the study were individuals ages 18 and up who agreed to participate. The study excluded participants who had been on ART for less than one month, did not complete the depression and anxiety scales, were in a postpartum period, were diagnosed with severe mental health issues (e.g., schizophrenia) or were unable to communicate with the interviewer.

### Sampling Method

The sample size was determined based on a single population proportion formula: $n=\frac{{Z_{1-\frac{\alpha }{2}}}^{2}P\left(1-P\right)}{d^{2}}$, in which ${Z_{1-\frac{\alpha }{2}}}^{2}$ is the confidence level, at 1.96; is depression prevalence, at 33.5% and is the margin of error, at 0.05. Assuming a nonresponse rate of 5%, the total required sample size was 361 participants. During the sampling process, 382 individuals were invited to participate, with 369 ultimately consenting to join the study (96.5%). The study adopted a convenience sampling method, with researchers engaging patients as they waited for their appointments or collected test results, carefully selecting participants based on the study’s inclusion and exclusion criteria. The researchers thoroughly explained the study to participants, addressed their questions and assured them that declining to participate would not impact their access to care at the clinic. All participants provided signed informed consent.

### Data Collection Procedure and Tools

Data collection was conducted through structured, face-to-face interviews with participants, using a prepared questionnaire that addressed key areas: demographic information; HIV diagnosis; ART treatment; risk behaviours and assessments of anxiety, depression, perceived social support and HIV-related stigma.

In this study, depression was assessed using the Patient Health Questionnaire-9 (PHQ-9) scale, which has demonstrated high reliability among the Vietnamese PLWHA population, with a Cronbach’s α of 0.91 (22). The PHQ-9 has a total score range of 0 to 27, with a cut-off of 5, indicating the presence of depressive symptoms, and a score of 10 or higher indicating clinically significant depression (23, 24). For our analysis, participants were classified as experiencing depression if their PHQ-9 score met or exceeded the clinical cut-off of 5.

Anxiety was evaluated using the Generalised Anxiety Disorder 7 (GAD-7) scale, which also has demonstrated high reliability in Vietnam, with a Cronbach’s α of 0.91 (25). The GAD-7 scores range from 0 to 21, with a cut-off of five indicating initial anxiety symptoms, and a score of 10 or above indicating clinically significant anxiety (26). In this study, individuals with a GAD-7 score of five or higher were identified as experiencing anxiety.

For social support, the Multidimensional Scale of Perceived Social Support (MSPSS) was used. This 24-item scale measures perceived support from family, friends and significant others and is highly reliable, with a Cronbach’s α of 0.9 overall, between 0.89 and 0.92 across subscales (27).

The HIV Stigma Short Form, developed by Reinius (28), assesses perceived stigma across four domains: Personalised Stigma; Disclosure Concerns; Negative Self-Image and Public Attitudes. Scores range from 12 to 48, with higher scores indicating stronger perceptions of stigma. The scale is reliable, with a Cronbach’s α above 0.7 for all domains, validating its utility for HIV populations (28).

### Data Analysis Methods

After data collection, the interviewer reviewed all questionnaires to identify any missing data or errors and finally excluded incomplete or incorrect responses from the analysis. Data were entered into Epidata (Version 4.6) and analysed using STATA (Version 17.0), with all datasets thoroughly cleaned and prepared for analysis.

Descriptive statistics – including frequencies, percentages, means, and standard deviations for normally distributed data or medians and interquartile ranges for skewed distributions – were used to summarise study variables.

The study used proportions to summarise the data and applied chi-square and Fisher’s exact tests to assess the relationship between participant characteristics and the presence of depressive and anxiety symptoms. The association between study factors and positive screening for depressive and anxiety symptoms was examined using univariate logistic regression.

Variables with a *P*-value < 0.2 in the univariate analysis were included in the multivariate logistic regression model to control for potential confounders. Initially, variables were retained in the model if *P* < 0.05, and subsequently, non-significant variables were reintroduced to assess their individual contributions. Logistic regression assumptions were checked, and model fit was evaluated using the Hosmer-Lemeshow goodness-of-fit test, with a *P*-value > 0.05 indicating an acceptable fit.

Adjusted odds ratios (AORs) with 95% confidence intervals (CIs) were reported to measure associations, with statistical significance set at *P* < 0.05.

### Ethical Approval

The study received ethical approval from the Biomedical Research Ethics Committee of the University of Medicine and Pharmacy at Ho Chi Minh City, under approval number 420/HDDD – DHYD, with code 24175 – DHYD, signed on 7 March 2024.

This study was reported in accordance with the Strengthening the Reporting of Observational Studies in Epidemiology (STROBE) guidelines for cross-sectional studies.

The study’s participants were informed about the research’s objectives and significance, as well as their right to withdraw from the study at any time or refrain from answering any questions they did not wish to answer. They provided written consent by signing the research information form. The study did not impact the participants’ physical and mental health, nor did it affect their treatment process.

## Results

### Participants’ Sociodemographic Characteristics

The study participants’ sociodemographic characteristics are as follows: 82.4% were male, and 71.8% were aged 19–39, with a mean age of 33.35 ± 9.80 years. Most participants (94.6%) were employed, and 86.2% self-reported being economically stable. Furthermore, 41.5% had attained at least a high school education, 73.7% lived with others, and 45.5% had a normal weight classification, according to WHO’s criteria for the Asia-Pacific population, with a mean BMI of 21.77 ± 3.47 kg/m^2^ (Table 1).

### Participants’ HIV Clinical and Risk Behaviour Characteristics

The majority of participants (70.7%) disclosed their HIV status to at least one person. Regarding the source of infection, 57.5% were unaware of how they contracted HIV. The median duration since initiating ART was four years (interquartile range: two to six years), with 59.8% having received treatment for one to five years. Only 2.7% reported experiencing side effects. Treatment adherence was observed in approximately 78.0% of participants.

The median CD4 cell count among participants was 266 cells/mm^3^ (range: 34–476 cells/mm^3^), with 42.2% having a count below 200 cells/mm^3^. Viral load data revealed that 83.7% of participants had levels below the detection threshold (Table 2).

In terms of substance use, 32.5% reported consuming alcohol, while 13.0% used tobacco. Regarding sexual behaviour, 49.3% reported engaging in sexual activity with male partners and 48.8% with female partners. Consistent condom use during sexual activity was reported by 36.6% of participants. A small proportion of participants had sought psychiatric evaluation or psychological counselling (3.8%), used medication for mental disorders (1.9%) or had a family history of mental health issues (0.5%) (Table 2).

Participants reported a mean social support score of 4.03 ± 0.98. Most participants (91.4%) reported receiving moderate to high levels of social support. However, perceived HIV stigma was prevalent, with 63.4% of participants reporting high stigma levels and a mean stigma score of 26.67 ± 6.76 (Table 2).[Table t3-11mjms3203_oa]

### Prevalence of Anxiety and Depression Among PLWHA

The findings reveal that 23.0% (95% Cl: 18.8–27.6) of participants exhibited symptoms of depression, with the majority experiencing mild depression (16.8%). Moderate depression accounted for 4.9%, while severe and very severe depression were the least prevalent, at 0.8% and 0.5%, respectively. Most participants (76.9%) did not present symptoms of depression.

Regarding anxiety disorders, 29.3% (95% CI: 24.6–34.1) presented symptoms, with mild anxiety being the most common (16.3%), followed by moderate anxiety (10.3%) and severe anxiety (2.7%). The remaining 70.7% of participants reported no symptoms of anxiety.

### Risk Factors Associated with Depression Among PLWHA in South Vietnam

The results from the univariate logistic regression analysis indicated that depression among PLWHA was associated with employment status, economic status, disclosure of HIV status and anxiety levels. Variables with *P*-values less than 0.2 in the univariable logistic regression analysis were included in the multivariable logistic regression analysis.

Sociodemographic characteristics, HIV clinical factors and risk behaviour characteristics – including gender, age group, education level, living status, BMI classification, HIV transmission mode, time since ART treatment, treatment adherence, viral load, CD4 count at treatment initiation, tobacco and alcohol use, condom use during sexual activity, sexual orientation, psychiatric examination/psychological counselling, family history of mental health issues, use of antidepressants/anxiolytics, perceived social support and HIV stigma – were not found to be statistically significant in association with depression among PLWHA in the univariable logistic regression analysis (*P* > 0.05).

The Hosmer-Lemeshow test suggests that the model fits the data well, with *P* = 0.49, indicating no significant difference between observed and predicted values.

The multivariable analysis identified anxiety levels as significant determinants of depression among PLWHA (Table 4). Anxiety levels made a strong and statistically significant impact on depression: Patients with severe anxiety were 100 times more likely to experience depression (AOR = 101.61, 95% CI: 11.67–884.28, *P* < 0.001), and moderate anxiety increased the risk more than 50 times (AOR = 58.06, 95% CI: 22.27–151.40, *P* < 0.001). These results highlight the close relationship between anxiety and depression, underscoring the need for integrated mental health interventions.

### Risk Factors Associated with Anxiety Among PLWHA in South Vietnam

The univariate logistic regression analysis identified several factors associated with anxiety disorders among PLWHA, including employment status, economic status, use of antidepressants or anxiolytics, treatment side effects, HIV stigma and receiving professional help from psychologists/psychiatrists.

Other sociodemographic characteristics, HIV clinical factors and risk behaviour characteristics were not found to be statistically significant in association with anxiety among PLWHA in the univariable logistic regression analysis (*P* > 0.05).

The Hosmer-Lemeshow test indicated that the model fit the data well, at *P* = 0.79, suggesting no significant difference between observed and predicted values.

The multivariate analysis revealed that experiencing side effects during treatment and perceiving high levels of HIV stigma were significant determinants of anxiety disorders among PLWHA. Specifically, patients who experienced side effects during treatment had an increased risk of anxiety disorders compared with those who did not (AOR = 5.92, 95% CI: 1.54–22.7, *P* = 0.01). Similarly, patients who perceived high levels of HIV stigma had an increased risk of anxiety disorders compared with those with low stigma perception (AOR = 2.51, 95% CI: 1.45–4.33, *P* = 0.001).[Table t5-11mjms3203_oa]

## Discussion

### The Mental Health Burden of Anxiety and Depression Among PLWHA: Prevalence Insights

This study highlights the significant mental health burden faced by PLWHA, with 23.0% of participants experiencing depressive symptoms, according to the PHQ-9 scale. This prevalence is comparable to rates reported in Vietnam, such as Bach’s study (20.2%), but lower than Thai’s findings (36.5%) (7, 29). Internationally, the depression prevalence observed in our study is lower than that of Ethiopia (41.7%) and Somalia (33.5%), yet higher than in China (14.0%) (6, 10, 30). These differences may be attributed to variations in assessment tools, cultural perceptions of mental health and study populations. For example, in our study, 93% of participants had been living with HIV for over a year, potentially developing coping mechanisms that mitigated depressive symptoms. However, depression’s persistent prevalence highlights the need for proactive mental health screening and support in HIV care settings.

Anxiety was similarly prevalent, with 29.3% of participants reporting symptoms on the GAD-7 scale, most of which were mild (16.3%). These rates are higher than those reported in some studies in Vietnam and similar contexts (31, 32) but lower than findings from systematic reviews, such as Lu Niu’s in China (> 40%) (17). Prevalence differences can stem from the use of various assessment tools, such as the HAM-A, which employs different thresholds for anxiety severity, as well as the diverse social and clinical contexts of PLWHA. Nevertheless, the high prevalence of anxiety underscores the dual burden of mental health challenges in this population, emphasising the importance of integrated mental health interventions in HIV care programmes.

### Emerging and Context-Specific Determinants of Mental Health Among PLWHA in South Vietnam

The univariate analysis found that financial status was associated with depression, but this relationship was not significant in multivariate analysis, contrasting with findings from studies in Africa and China, in which financial stress was a significant determinant (6, 10, 30). This may reflect our study population’s unique characteristics, as most participants had lived with HIV for over a year, disclosed their status (70.7%) and reported medium or high social support (91.4%) – factors that may reduce the psychological impact from financial stress.

No significant associations were found between sociodemographic factors and depression, suggesting that depression among PLWHA may be influenced more by psychological than by external factors, such as socioeconomic status (33). Depression among PLWHA can become chronic, driven by complex factors such as stigma, coping mechanisms and social support (34). The literature, including meta-analysis, has highlighted that while low socioeconomic status is often linked to higher psychiatric morbidity, this association may be less pronounced among PLWHA (35). These findings emphasise the need for holistic interventions that address resilience and social dynamics to manage chronic depressive symptoms in this population more effectively.

Furthermore, the study found a significant statistical difference between levels of anxiety disorders and depression, aligning with extant studies (8). This may be explained by how prolonged anxiety can lead to increasing anxiety levels, which can lead to depression. Therefore, both groups of symptoms, whether related to anxiety disorders or depression, are psychological manifestations that can impact their health and quality of life negatively.

The study also found that individuals experiencing side effects from ART medication during treatment had a higher risk of anxiety disorders than those who did not experience such side effects. The results are consistent with a study by Abadiga, which also noted that individuals with adverse drug reactions had more depressive symptoms than those without such reactions (10, 30). This can be explained by how undesirable side effects from ART medication can disrupt normal activities among PLWHA, leading to increased feelings of frustration and a higher prevalence of anxiety symptoms.

We also found a correlation between anxiety disorders and perception of HIV stigma. Patients in the group with a high perception of HIV stigma had a higher risk of anxiety disorders than those in the group with a low perception of HIV stigma. This result is consistent with previous studies (10, 30). One possible explanation is that HIV is a chronic and transmissible disease and that the perception of stigma is influenced by local culture, leading patients to prefer isolation to avoid stigma, which can contribute to the development of depression. Therefore, living in a trustworthy social environment with a lower stigma towards PLWHA helps reduce daily stress and protects against mental health issues.

## Conclusion

This study highlights mental health challenges among PLWHA in South Vietnam, with 23.0% experiencing depression and 29.3% presenting anxiety. Anxiety was found to be a key factor influencing depression, with severe anxiety increasing the risk. These results point to the importance of addressing co-occurring mental health conditions in HIV care. Unlike findings in other contexts, socioeconomic and sociodemographic factors were not associated significantly with depression, possibly due to high levels of social support and clinical stability in this population. However, anxiety disorders were linked to treatment side effects and high levels of HIV stigma, highlighting the importance of reducing stigma and managing medication-related issues in HIV care.

This study provides new evidence concerning mental health issues and related risk factors among PLWHA in South Vietnam. The findings can guide the development of focused and culturally appropriate interventions to improve this population’s mental health and quality of life.

## Figures and Tables

**Table 1:** Participants’ sociodemographic characteristics (*n* = 369)

Characteristics		Frequency (%)
Gender	Male	304 (82.4)
	Female	65 (17.6)

Age group	19–39	265 (71.8)
	40–49	79 (21.4)
	≥ 50	25 (6.8)

Employment status	Permanent job	280 (75.9)
	Temporary job	69 (18.7)
	Unemployed	20 (5.4)

Economic status	Financially struggling	51 (13.8)
	Stable income	314 (85.1)
	Well-off	4 (1.1)

Education level	Below primary school/unschooled	16 (4.3)
	Primary school	25 (6.8)
	Secondary school	69 (18.7)
	High school	106 (28.7)
	Bachelor’s (or higher)	153 (41.5)

Living status	Living alone	97 ( 26.3)
	Living with a spouse/partner	114 (30.9)
	Living with friends	21 (5.7)
	Living with relatives/family	137 ( 37.1)

BMI classification	Underweight (BMI < 18.5 kg/m^2^)	71 (19.2)
	Normal weight (18.5 ≤ BMI < 23 kg/m^2^)	168 (45.5)
	Overweight (23 ≤ BMI < 25 kg/m^2^)	67 (18.2)
	Obese (BMI ≥ 25 kg/m^2^)	63 (17.1)

**Table 2:** Participants’ HIV clinical and risk behaviour characteristics (*n* = 369)

Characteristics		Frequency (%)
HIV disclosure	Yes	261 (70.7)
	No	108 (29.2)

HIV transmission	Sexual transmission	126 ( 34.1)
	Blood transmission	31 (8.4)
	Unknown source of transmission	212 (57.5)

Time since ART treatment (years)	< 1 Year	27 (7.3)
	1–5 Years	221 (59.8)
	> 5 Years	121 (32.8)

Experienced side effects during treatment	Yes	10 (2.7)
	No	359 (97.3)

Treatment adherence	Yes	289 (78.0)
	No	80 (22.0)

Viral load	Below detection threshold	309 (83.7)
	Above detection threshold	60 (16.3)

CD4 count at treatment initiation (*n* = 282)	< 200	119 (42.2)
	200–349	51 (18.1)
	350–499	48 (17.0)
	≥ 500	64 (22.7)

Tobacco	Yes	48 (13.0)
	No	321 (87.0)

Alcohol	Yes	120 (32.5)
	No	249 (67.5)

Condom use during sexual activity	Yes	135 (36.6)
	No	234 (63.4)

Sexual orientation	Males	182 (49.3)
	Females	180 (48.8)
	Both	7 (1.9)

Psychiatric examination/psychological counselling	Yes	14 (3.8)
	No	355 (96.2)

Family members with mental health issues	Yes	2 (0.5)
	No	367 (95.5)

Use of antidepressants/anxiolytics	Yes	7 (1.9)
	No	362 (98.1)

Perceived social support	Low	32 (8.7)
	Medium	291 (78.9)
	High	46 (12.5)

HIV stigma	Low	135 (36.6)
	High	234 (63.4)

**Table 3 t3-11mjms3203_oa:** Prevalence of anxiety and depression among PLWHA (*n* = 369)

Mental health conditions		Frequency (%)
Depression	Yes	85 (23.0)
	No	284 (77.0)

Severity of depression	No depression	284 (77.0)
	Mild depression	62 (16.8)
	Moderate depression	18 (4.9)
	Severe depression	3 (0.8)
	Very severe depression	2 (0.5)

Anxiety disorder	Yes	108 (29.3)
	No	261 (70.7)

Severity of anxiety disorder	No anxiety	261 (70.7)
	Mild anxiety	60 (16.3)
	Moderate anxiety	38 (10.3)
	Severe anxiety	10 (2.7)

**Table 4:** Multivariable analysis of factors associated with depression (*n* = 369)

Characteristics (*n* = 369)		Depression		COR (95% Cl)	*P*	AOR (95% Cl)	*P*

		Yes (*n* = 85)	No (*n* = 284)				
Employment status	Permanent job	54 (19.3)	226 (80.7)	1		1	
	Temporary job	22 (31.9)	47 (68.1)	0.67 (0.08–1.25)	0.025*	1.88 (0.83–4.23)	0.125
	Unemployed	9 (45.0)	11 (55.0)	1.23 (0.30–2.16)	0.009*	2.99 (0.81–11.03)	0.099

Economic status	Stable income	62 (19.8)	252 (80.2)	1		1	
	Well-off	3 (75.0)	1 (25.0)	2.50 (0.22–4.78)	0.003*	15.7 (1.00–247.67)	0.050
	Financially struggling	20 (39.2)	31 (60.8)	0.96 (0.33–1.59)	0.032*	1.14 (0.43–3.01)	0.786

HIV disclosure	No	32 (29.6)	76 (70.4)	1		1	
	Yes	53 (20.3)	208 (79.7)	1.65 (0.99–2.75)	0.054	1.31 (0.65–2.62)	0.438

Severity of anxiety disorder	No anxiety	20 (7.7)	241 (92.3)	1		1	
	Mild	25 (41.7)	35 (58.3)	2.15 (1.46–2.83)	< 0.001*	7.82 (3.83–15.99)	< 0.001*
	Moderate	31 (81.6)	7 (18.4)	3.97 (3.03–4.91)	< 0.001*	58.06 (22.27–151.40)	< 0.001*
	Severe	9 (90.0)	1 (10.0)	4.68 (2.57–6.80)	< 0.001*	101.61 (11.67–884.28)	< 0.001*

* *P* < 0.05

**Table 5 t5-11mjms3203_oa:** Multivariable analysis of factors associated with anxiety (*n* = 369)

Characteristics (n = 369)		Anxiety		COR (95% Cl)	*P*	AOR (95% Cl)	*P*

		Yes (*n* = 108)	No (*n* = 261)				
Employment status	Permanent job	74 (26.5)	206 (73.5)	1		1	
	Temporary job	24 (34.7)	45 (65.3)	1.48 (0.84–2.60)	0.168	1.22 (0.65–2.27)	0.522
	Unemployed	10 (50.0)	10 (50.0)	2.78 (1.11–6.95)	0.028*	1.76 (0.57–5.37)	0.319

Economic status	Stable income	83 (26.4)	231 (73.6)	1		1	
	Well-off	2 (50.0)	2 (50.0)	2.78 (0.38–20.07)	0.310	2.51 (0.27–23.02)	0.413
	Financially struggling	23 (45.1)	28 (54.9)	2.28 (1.24–4.19)	0.007*	1.80 (0.89–3.66)	0.100

Use of antidepressants or anxiolytics	Yes	6 (85.7)	1 (14.3)	2.72 (0.59–4.85)	0.012*	3.19 (0.21–48.33)	0.402
	No	102 (28.2)	260 (71.8)	1		1	

Experienced side effects during treatment	Yes	6 (60.0)	4 (40.0)	1.32 (0.04–2.61)	0.043*	5.92 (1.54–22.7)	0.010*
	No	102 (28.4)	257 (71.6)	1		1	

HIV stigma	High	81 (34.6)	153 (65.4)	2.11 (1.28–3.49)	0.003*	2.51 (1.45–4.33)	0.001*
	Low	27 (20.0)	108 (80.0)	1		1	

Psychiatric examination or psychological counselling	Yes	10 (71.4)	4 (28.6)	1.88 (0.69–3.06)	0.002*	4.51 (0.90–22.55)	0.066
	No	98 (27.6)	257 (73.4)	1		1	

Note:

* *P* < 0.05
